# Improved Performance of Perovskite Light-Emitting Diodes by Quantum Confinement Effect in Perovskite Nanocrystals

**DOI:** 10.3390/nano8070459

**Published:** 2018-06-25

**Authors:** Lung-Chien Chen, Zong-Liang Tseng, Dai-Wei Lin, Yu-Shiang Lin, Sheng-Hui Chen

**Affiliations:** 1 Department of Electro-optical Engineering, National Taipei University of Technology, 1, 3 Section, Chung-Hsiao East Road, Taipei 106, Taiwan; t104658068@ntut.org.tw (D.-W.L.); t104658044@ntut.org.tw (Y.-S.L.); 2 Department of Optics and Photonics, National Central University, 300 Chung-Da Rd., Chung-Li 32001, Taiwan; ericchen@dop.ncu.edu.tw

**Keywords:** PeLEDs, OAB, perovskite, quantum confinement effect

## Abstract

In this study, we demonstrate an easy and reliable solution-processed technique using an extra adductive in the perovskite precursor solution. Using this method, a dense and uniform morphology with full surface coverage and highly fluorescent films with nanoscale crystal grains can be obtained. The high exciton binding energy in the resulting films employing octylammonium bromide (OAB) adductives proved that high fluorescence originated from the quantum confinement effect. The corresponding perovskite light-emitting diodes (PeLEDs) that were based on this technique also exhibited excellent device performance.

## 1. Introduction

Since the report on the perovskite light-emitting diodes (PeLEDs) in 2012 [1] expanded the research range of lead halide perovskites from their photovoltaic applications, a series of studies on device structures and deposition methods [2,3,4,5,6,7,8,9,10] have been presented. As a result, excellent electroluminescence efficiency of 42.9 cd/A has been achieved by employing an additive-based nanocrystal pinning technique [11].

**Figure 1:**
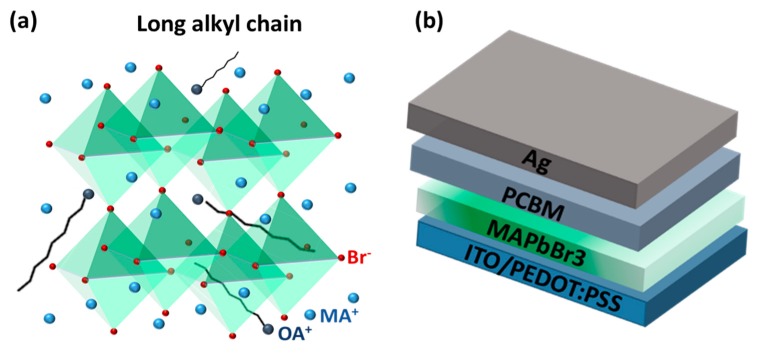
(**a**) Illustration for substituting octylammonium (OA^+^) for methylammonium (MA^+^) in the MAPbBr_3_ array; (**b**) Schematic of the device structure of perovskite light-emitting diodes (PeLEDs) in this study. PCBM, [6,6]-phenyl-C61-butyric acid methyl ester. PEDOT:PSS, poly(3,4-ethylenedioxythiophene) polystyrene sulfonate. ITO, indium tin oxide.

The active layer of PeLEDs and CH_3_NH_3_PbBr_3_ (MAPbBr_3_) can be prepared by using a simple solution-processed coating, where a precursor solution containing CH_3_NH_3_Br (MAB) and PbBr_2_ is spin-coated on the substrates and washed using toluene to rapidly crystalize while spinning [12,13]. The resulting film exhibited better optoelectronic properties, such as high mobility [14,15,16,17], long and balanced electron-hole diffusion lengths [18,19], low bulk defect densities, and slow Auger recombination [20] compared to the previous solution-processed semiconductors. On the other hand, Pérez-Prieto et al. [21,22] first reported that MAPbBr_3_ perovskite quantum dots (QDs) were achievable and they synthesized them using octylammonium bromide (CH_3_(CH_2_)_7_NH_3_Br; OAB) with a long alkyl chain as a capping ligand to stabilize them. The longer alkyl chain cations are embedded in the MAPbBr_3_ lattice to replace the methyl ammonium cations and their long chains dangling outside the lattice, as illustrated in Figure 1a. Because of the repulsion forces between long alkyl chains, the growth of the perovskite array is suppressed in three dimensions, resulting in perovskite QDs being produced. Although it is well known that colloidal QD dispersions exhibit narrow-band emission and high photoluminescence (PL) efficiency [21,22,23,24,25,26,27], the uniform, smooth, and large-area films are difficult to prepare by directly using colloidal dispersions. In addition, some solution-processed methods for highly efficient PeLEDs with high-quality perovskite films, such as the additive-based nanocrystal pinning technique, require great skill for processing controls. Therefore, it is necessary to develop an easy and reliable method for obtaining high-quality perovskite films with nanoscale crystal grains for high-performance PeLEDs [28,29,30].

In this study, we demonstrate the preparation of MAPbBr_3_ thin films with highly uniform and dense nanoscale grains through a simple spin-coating method using OAB as an additive (Figure 1a). Highly fluorescent thin films with full-surface coverage were achieved by optimizing the amount of OAB in the perovskite precursors. Highly efficient PeLEDs were also prepared using the resulting MAPbBr_3_ thin films as active layers. The champion device based on the OAB adductive method exhibited a maximum luminance of 310 cd/m^2^ (at 4.5 V) and a maximum luminous current efficiency of 1.21 cd/A (at 4.5 V). We further show that the improved performance of PeLEDs and the enhanced fluorescence of MAPbBr_3_ thin films are due to high exciton binding energy in nanometer-sized crystal grains, which leads to reduced nonradiative recombination and increased emission efficiency.

## 2. Materials and Methods 

PeLEDs with a device structure of glass/indium tin oxide (ITO)/poly(3,4-ethylenedioxythiophene) polystyrene sulfonate (PEDOT:PSS)/MAPbBr3/[6,6]-phenyl-C61-butyric acid methyl ester (PCBM)/Ag were used in this study, as illustrated in Figure 1b. The PEDOT:PSS layers using AI-4083 (Heraeus Clevios) were spin-coated on a cleaned ITO substrate at 5000 rpm for 30 s and were post-annealed at 120 °C for 10 min. PbBr_2_ (99.999%; Sigma-Aldrich, St. Louis, MO, USA), methylammonium iodide (MAB; Lumtec, Hsinchu, Taiwan), and octylammonium bromide (OAB; Lumtec, Hsinchu, Taiwan) were dissolved in a dimethyl sulfoxide (DMSO)/dimethylformamide (DMF) mixture (7:3, *v*/*v*) as the precursor, with an MAPbBr_3_ concentration of 0.5 M. Different OAB ratios were prepared with the weight ratio of MAB:OAB. The perovskite precursors were then spin-coated onto the PEDOT:PSS layers at 5000 rpm for 30 s, with 2 mL of anhydrous toluene dropped at 27 s during spin-coating. The large amount of toluene ensured the removal of OAB due to the long alkyl chain approaching nonpolar character. The as-deposited films were post-annealed at 90 °C for 5 min, and after being cooled to room temperature, [6,6]-phenyl-C61-butyric acid methyl ester (PCBM) dissolved in chlorobenzene (20 mg/mL) was then spin-coated on them at 1200 rpm for 30 s. Ag electrodes (100 nm) were evaporated through a metal mask to define the device area (0.1 cm^2^). 

The crystalline microstructure, absorbance spectra, and surface morphology of the films were determined by X-ray diffraction with Cu-Kα radiation (D8 Discover, Bruker, Karlsruhe, Germany), UV-visible spectroscopy (U-4100, Hitachi High-Technologies Co., Tokyo, Japan), and a field-emission scanning electron microscope (GeminiSEM, Zeiss, Oberkochen, Germany), respectively. The photoluminescence (PL) spectra were measured using an optical microscope-based system (UniRAM, Protrustech, New Taipei, Taiwan) with an excitation of 405 nm. The temperature-dependent photoluminescence (PL) spectra was measured under a nitrogen-filled atmosphere. The current density-voltage-luminesce (J-V-L) characteristics were measured using a Keithley 2400 combined with a SpectraScan Spectroradiometer (PR-670, Photo Research, New York, NY, USA). 

## 3. Results and Discussion

**Figure 2:**
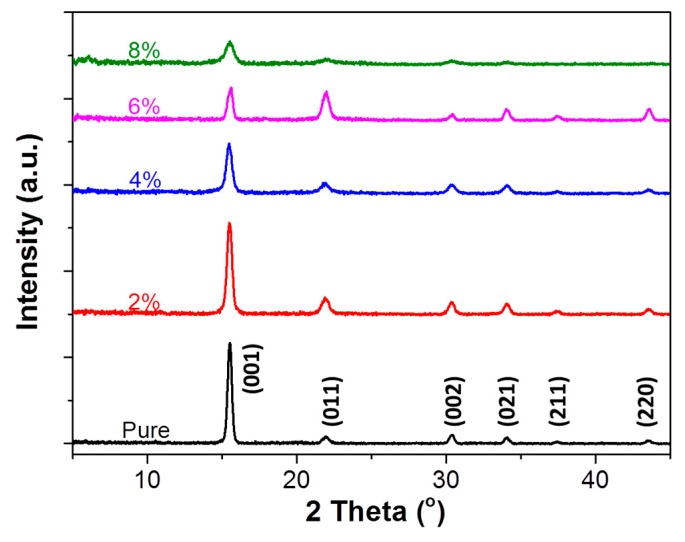
XRD patterns of the MAPbBr_3_ films with different octylammonium bromide (OAB) ratios deposited on the glass.

To understand how the ratio of OAB to MAPbBr_3_ in the precursor solution affects the performance of the resulting perovskite light-emitting diodes, X-ray diffraction patterns of MAPbBr_3_ films grown with different OAB ratios were collected, and these are shown in Figure 2 (the corresponding 2D-XRD is shown in Supplementary Figure S1). Pure MAPbBr_3_ cubic phase with (001), (011), (002), (021), (211), and (220) at 2θ range from 5–45° were identified [23] in all diffractograms. The thicknesses of the MAPbBr_3_ films that were obtained by α-step had no significant differences at different OAB ratios (~200 nm; Supplementary Figure S2). Using a higher OAB ratio, the intensity of the (001) peak decreased and full width at half maximum (FWHM) increased, implying a smaller MAPbBr_3_ grain size. Also, when the OAB ratio was increased to 6%, the intensity and FWHM ratio of the (001) to (110) peak decreased. Reduced crystallinity of MAPbBr_3_ with an OAB adductive compared to pure MAPbBr_3_ films suggests that the long alkyl chain of OAB indeed acts as a better capping ligand to limit MAPbBr_3_ grain growth. Therefore, the OAB ratio in the precursor solution was confirmed to suppress the grain growth in the MAPbBr_3_ films. Interestingly, when applying a higher OAB ratio (8%), MAPbBr_3_ became very weak and produced a new diffraction peak at a low angle (~6°). Most reported XRD patterns for MAPbBr_3_ were only detected from 10° (2θ > 10°), therefore it was difficult to know the exact components and structures of this new phase by only depending on XRD. Nevertheless, according to the Scherrer equation [30], we can obtain that the low-angle peak corresponded to a larger lateral ordered spacing. 

**Figure 3:**
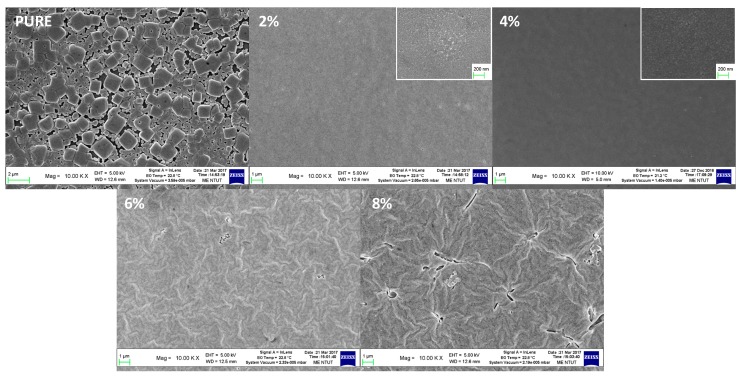
Scanning electron microscopy (SEM) images of the MAPbBr_3_ films with different OAB ratios deposited on the glass. The insets for 2% and 4% show images with higher magnification.

Furthermore, the OAB ratio also affected the surface morphology of the MAPbBr_3_ films, as revealed by scanning electron microscopy (SEM) micrographs, shown in Figure 3. The morphology of the pure MAPbBr_3_ films showed large and nonuniform cubic grains from 200 to 1000 nm, but some interspace existed between the micrograins, which may have increased leakage due to the direct contact between the upper and bottom layers without passing through the perovskite films. The unwanted broad size distribution was due to the rapid crystallization process in the perovskite thin films [31,32], leading to not enough time for the thermodynamically spontaneous process, i.e., the well-known Ostwald ripening process [33,34], to form small-size crystals that recrystallized to large grains. Several methods have been suggested to improve this phenomenon in perovskite thin films, such as the solvent-annealing process [35], mixed halide treatment [34], and HBr/DMF cosolvent [10]. Applying OAB as an additive, the grain size was significantly reduced, which was in accord with XRD results. Smooth, uniform, and dense perovskite films (grain size of about 20 nm) were observed when the OAB ratio was equal to 2% and 4%, and furthermore, the grain size of the 2% sample was more uniform than that of the 4% sample (see insets in Figure 3). This suggested that the OAB additive is the main factor in the formation of nanograins in perovskite film. This dense MAPbBr_3_ film was also reported using the HBr/DMF cosolvent method [10], which originated from slow crystallization rates during deposition. Unlike those, the reason in our case for morphological control was the grain growth being limited by the long alkyl chain of OAB. Therefore, our grain sizes were much smaller than theirs and were much like those using an additive-based nanocrystal pinning technique [11]. Under a higher OAB ratio, inhomogeneous humped structures could be seen, resulting in rough films with some cracks and defects, which negatively affected the device performance. The humped structure may be attributed to large-size ordered packing that was formed from the grain aggregation. This may be why the low angle phase was found in the XRD pattern. These results indicate that the film morphology of MAPbBr_3_ films is strongly influenced by the OAB ratio in the precursor solution. This easy method, using an OAB adductive, provides a general way to control the morphology and the surface coverage of MAPbBr_3_ films.

**Figure 4:**
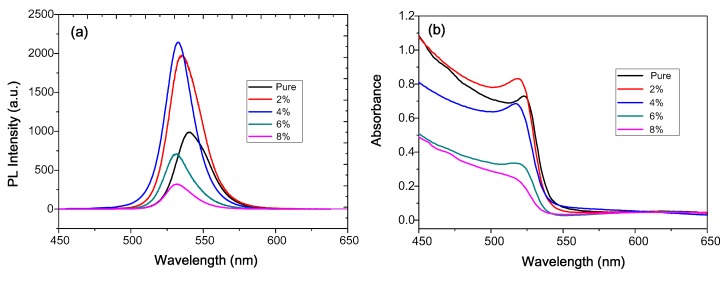
(**a**) PL spectra and (**b**) the absorbance of the MAPbBr_3_ films with different OAB ratios deposited on the PEDOT:PSS glass.

Figure 4a shows the PL spectra of samples with different OAB ratios in the precursor solution. The intensity increased using lower OAB ratios (2% and 4%), but decreased using low OAB ratios (6% and 8%). The PL intensity in the 4% sample is better than that in the 2% sample, which can be attributed to more homogeneous grain size (Figure 3). The weak PL in the 6 and 8% samples may be due to the poor crystallinity and grain aggregation, which may increase the dissociation rate. However, the PL intensity in thin films compared to that in dispersions is much more complicated [36,37] due to exciton dissociation between grains or bottom/upper layers, leading to radiative loss. Therefore, the smaller grains in the 2% and 4% films had more grain boundaries (more grain package) to provide PL quenching sites, but the 2% and 4% samples exhibited better PL intensities. The reason is that excitons are confined in the nanometer-sized grains [21,22], leading to strong PL emission. These results are similar to those using colloidal perovskite QD dispersions to directly deposit thin films [38,39,40,41]. An exciton diffusion length of 67 nm in MAPbBr_3_ films with nanograins was reported, which is much smaller than that of solution-processed perovskite films [11]. Moreover, a significant blue shift can be observed in PL spectra, in that emission wavelength decreased with an increase in the OAB ratio, from 540.1 nm (pure film) to 531.7 nm (8%). Similarly, the absorption edges in ultraviolet-visible (UV-vis) spectra (Figure 3b) also showed the same trend. The fitting bandgap (the corresponding Tauc plots shown in Supplementary Figure S3) from UV-vis spectra increased with an increase in the OAB ratio. The blue shift can typically be attributed to the quantum confinement effect in the nanocrystal [21,22]. Besides, nanocrystal materials have sharper density of states than higher dimensional materials. Therefore, they permit more electrons to occupy the states in conduction band, such that opportunity of spontaneous emission increases.

**Figure 5:**
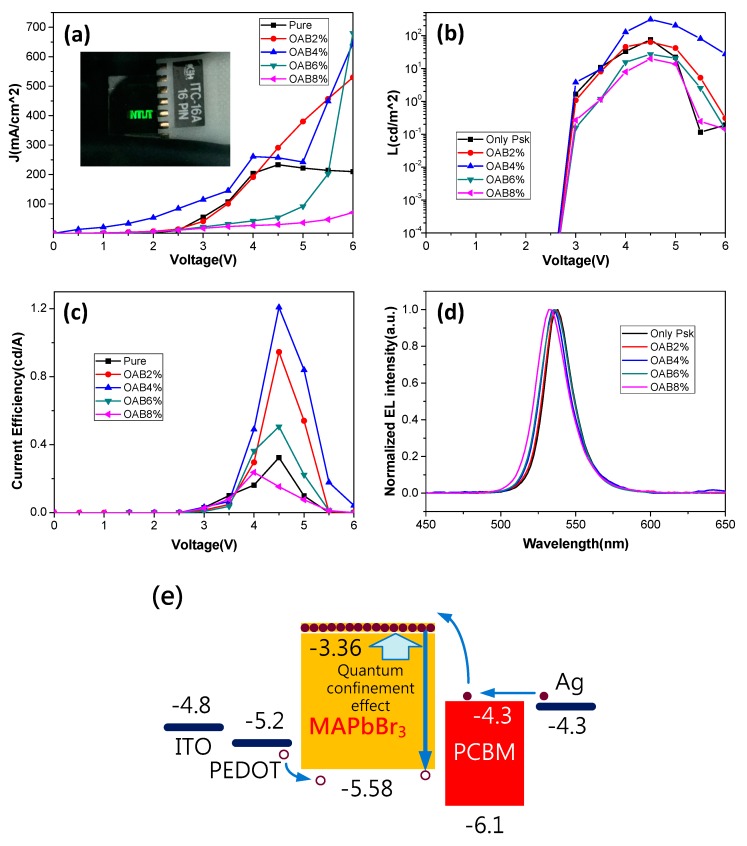
(**a**) Current–density vs. voltage, (**b**) luminance vs. voltage, (**c**) current efficiency vs. voltage, and (**d**) electroluminescence of PeLEDs based on MAPbBr_3_ films with different OAB ratios deposited on the glass. (**e**) a diagram of the energy band structure of the PeLEDs. The inset in (**a**) shows a photo image of our PeLED.

Figure 5a shows the current–density vs. voltage (J-V) curves for PeLEDs using different OAB ratios. All of the curves revealed diode behavior. The inserted image in Figure 5a shows that our PeLED displayed a text patterned by a metal mask for Ag evaporation. Figure 5b,c shows the luminance vs. voltage (L-V) and the current efficiency vs. voltage (CE-V) of our PeLEDs with different OAB ratios. The optimized PeLED that was prepared with the OAB ratio of 6% exhibited a maximum luminance of 310 cd/m^2^ (at 4.5 V) and a maximum luminous current efficiency of 1.21 cd/A (at 4.5 V). The PeLED that was based on the pure MAPbBr_3_ film without OAB adductives showed poor luminance characteristics (maximum CE = 0.32 cd/A), mainly due to high leakage current, as mentioned in Figure 3, that was induced from the lateral space between the MAPbBr_3_ grains or the pinholes on the film surface. Maximum current efficiency was achieved (1.21 cd/A) when the OAB ratio was increased to 4%. The excellent performance at the OAB ratio of 4% was mainly due to the dense and uniform MAPbBr_3_ layer with full coverage avoiding leakage, the smooth surface providing good contact with the electrode transporting layer (PCBM), and the MAPbBr_3_ nanograins having an enhanced radiative recombination rate for injected carriers. In contrast, poor device performance at higher OAB ratios (6% and 8%) may have been from the grain aggregation, leading to an increased dissociation path and a rough film surface. Furthermore, the electroluminescence (EL) spectra also exhibited a blue shift (Figure 5d), which was consistent with the PL observation. It is worth mentioning that the maximum luminance and current efficiency values of our PeLED are comparable to those of the previously reported PeLEDs based on MAPbBr_3_ active layers [2,3,4,5,6,7,8,9,10]. Figure 5e shows the energy band structure to explain the mechanism of the radiative recombination caused by the quantum confinement effect, as results of the blue shift and enhance the spontaneous emission of the electroluminescence (EL) spectra. In Figure 5, the phenomenon of luminance decay at high applied voltage is caused either by heat due to the series resistance of the devices or by damage due to the high electrical field.

**Figure 6:**
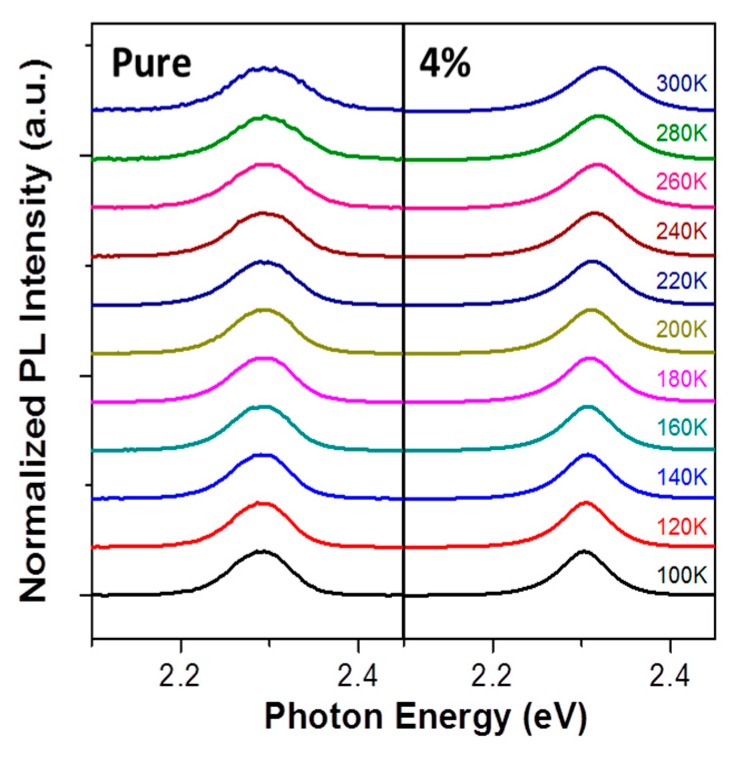
Temperature-dependent photoluminescence spectra for MAPbBr_3_ film with an OAB ratio of 4% and without OAB.

To better realize the nature of strong PL emission in the MAPbBr_3_ nanograins formed by the OAB adductive, the excitonic characteristics of the MAPbBr_3_ films should be considered. As we reported previously [42], temperature-dependent photoluminescence (TDPL) can be used to determine the exciton binding energy by linear fitting with a PL spectral broadening equation [43] at different temperatures:

ln(hΔν − hΔν_0_) = ln(hν_T_) − E_b_/K_B_T (1)

where hΔν is the full width at half maximum (FWHM) of the PL spectrum at some temperature, hΔν_0_ is the FWHM of the PL spectrum at the initial temperature, hν_T_ is related to the thermal dissociation rate, E_b_ is the exciton binding energy, K_B_ is the Boltzmann constant, and T is the temperature. The PL spectra at temperatures from 100 to 300 K for the MAPbBr_3_ films deposited with (4%) and without (pure) the OAB adductive are shown in Figure 6. The peak of both samples significantly broadened with increased temperature due to exciton–phonon interaction [17]. Grätzel et al. suggested that the dual PL emissions in MAPbBr_3_ at temperatures below 175 K were due to the coexistence of MA-ordered and MA-disordered domains in the MAPbBr_3_ array [44]. However, only one peak of both samples can be found in Figure 6. Indeed, in our case, linear fitting could not be estimated (very low R-square) if the temperature selected was from 100 to 300 K. Therefore, the temperature was set from 180 to 300 K for linear fitting in Supplementary Figure S4. R-square over 0.99 reveals a good fit, and exciton binding energies of 48 and 85 meV were determined for pure MAPbBr_3_ film and film using the OAB adductive, respectively. The higher exciton binding energy of the MAPbBr_3_ film with OAB compared to that of the pure MAPbBr_3_ film indicates strong exciton localization, which can block the exciton dissociation and increase the radiative recombination rate. Therefore, this provides evidence for the quantum confinement effect in nanometer-sized grains of MAPbBr_3_ film using the OAB adductive.

## 4. Conclusions

In conclusion, we have demonstrated that the preparation method employing OAB adductive can obtain high-quality MAPbBr_3_ thin films. The addition of a small amount of OAB in the MAPbBr_3_ precursor solvent produces a dense and uniform film morphology with full surface coverage. Also, it enables the formation of nanoscale MAPbBr_3_ grains, which enhance PL emission due to the quantum confinement effect. The high exciton binding energy in the MAPbBr_3_ films that are formed by the OAB adductive is evidence for the quantum confinement effect in the nanometer-sized grains, which reduces exciton dissociation and enhances exciton radiation. The quantum confinement effect also affects bandgap, which shifts to a short wavelength with an increasing OAB ratio. A blue shift is found in absorbance, PL, and EL spectra at different OAB ratios. The optimized PeLED with an OAB ratio of 4% exhibited a maximum luminance of 310 cd/m^2^ (at 4.5 V) and a maximum luminous current efficiency of 1.21 cd/A (at 4.5 V). This study offers a promising approach for reliably depositing high-quality MAPbBr_3_ thin films for application to efficient PeLEDs.

## Supplementary Materials

The following are available online at http://www.mdpi.com/2079-4991/8/7/459/s1, Figure S1: Original 2D XRD data of perovskite films with different OAB ratios, Figure S2: Film thicknesses of perovskite films with different OAB ratios. The error bars are standard deviations obtained from five samples, Figure S3: The corresponding Tauc plots from UV-vis spectra for linear fitting to determine bandgaps of perovskite films with different OAB ratios. The solid lines are fittings, Figure S4: Temperature-dependent data for linear fitting exciton binding energy. The solid lines are fittings.
