# Supplementary material for: Improved Performance of Perovskite Light-Emitting Diodes by Quantum Confinement Effect in Perovskite Nanocrystals
Source: Nanomaterials (Basel). 2018 Jun 25;8(7):459. doi: 10.3390/nano8070459 (PMC6071281; doi:10.3390/nano8070459)
Supplement: Supplementary file 1 [file nanomaterials-08-00459-s001.docx]

**Supporting Information**


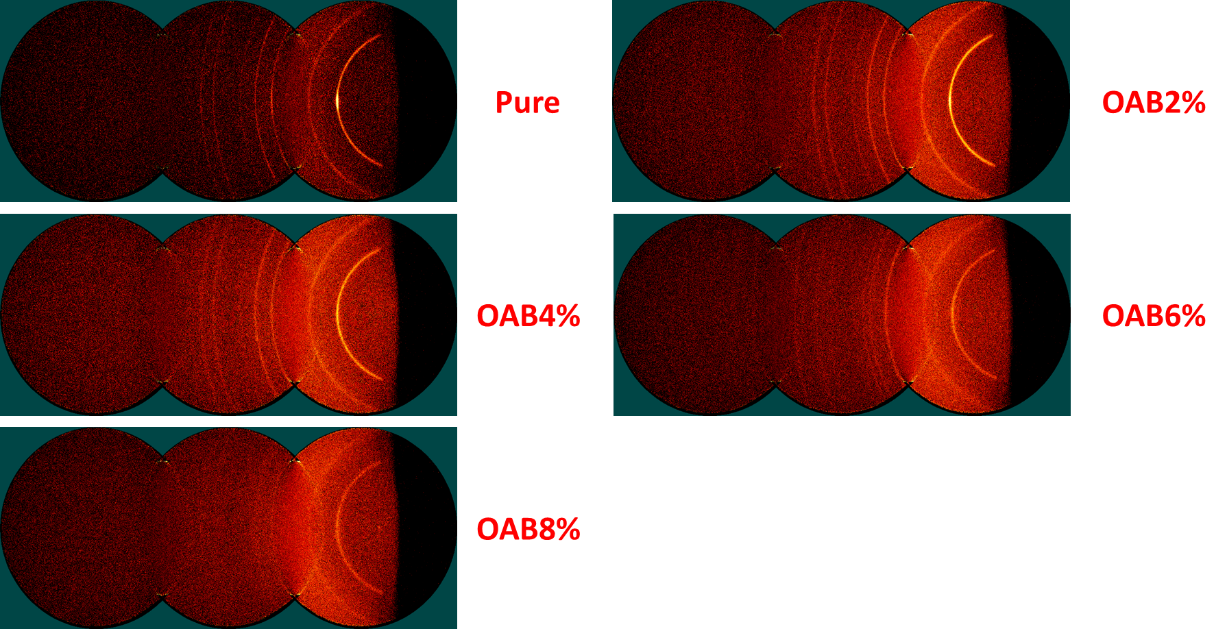


**Figure S1.** Original 2D XRD data of perovskite films with different OAB ratios.


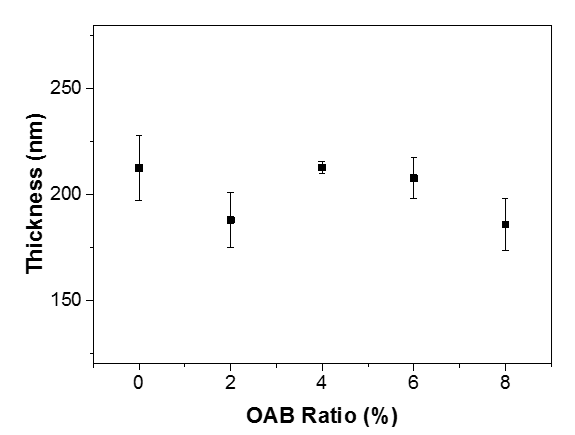


**Figure S2.** Film thicknesses of perovskite films with different OAB ratios. The error bars are standard deviations obtained from five samples.


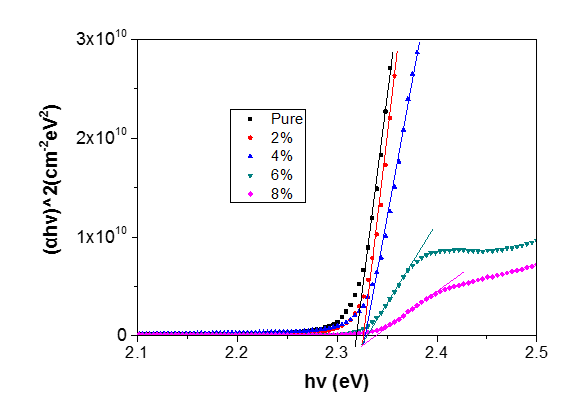


**Figure S3.** The corresponding Tauc plots from UV-vis spectra for linear fitting to determine bandgaps of perovskite films with different OAB ratios. The solid lines are fittings.


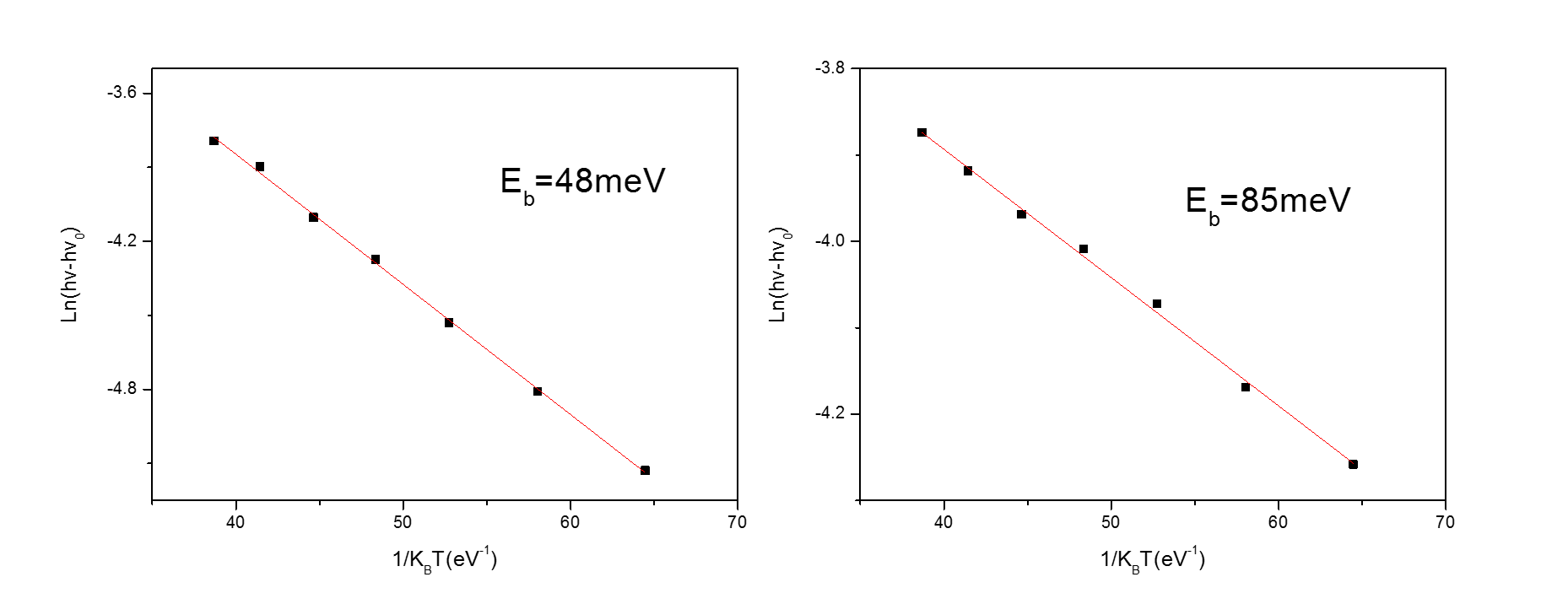


**Figure S4.** Temperature-dependent data for linear fitting exciton binding energy. The solid lines are fittings.
